# A Case of Cryoglobulinemic Vasculitis: Linking Thymectomy, Autoimmune Disease, and Lymphomagenesis

**DOI:** 10.1016/j.xkme.2026.101303

**Published:** 2026-02-12

**Authors:** Zainab Hameed, Nicholas Carlson, Mads Hornum, Nina Loeth Maartensson, Karl Emil Nelveg-Kristensen

**Affiliations:** 1 Department of Nephrology, Copenhagen University Hospital – Rigshospitalet, Copenhagen, Denmark; 2 Department of Pathology, Copenhagen University Hospital – Rigshospitalet, Copenhagen, Denmark

**Keywords:** Autoimmune disease, B-cell clonal evolution, cryoglobulinemic vasculitis, immune complex glomerulonephritis, marginal zone lymphoma, renal failure, thymectomy

## Abstract

This case illustrates the possible interplay between autoimmunity, chronic antigenic stimulation, cryoglobulinemic vasculitis, and lymphoproliferative disorders. The patient’s progression from myasthenia gravis and total thymectomy to systemic lupus erythematosus, cryoglobulinemic vasculitis, and marginal zone lymphoma demonstrates a continuum of immune dysregulation. Total thymectomy may impair central tolerance, predisposing to secondary autoimmunity and chronic B-cell activation. A 39-year-old woman presented with acute kidney injury, nephrotic-range proteinuria, and pulmonary edema. Laboratory evaluation revealed hypocomplementemia, positive cryoglobulins, a monoclonal IgM band, and elevated rheumatoid factor, findings consistent with cryoglobulinemic vasculitis and immune complex activity. The kidney biopsy demonstrated immune complex-mediated pseudothrombi. She was managed with high-dose corticosteroids, continuous renal replacement therapy, and rituximab, resulting in recovery of renal and cardiac function. During follow-up, she experienced a relapse of cryoglobulinemic vasculitis requiring further rituximab, after which renal function stabilized. This case highlights the complex relationship between thymectomy, autoimmunity, cryoglobulinemic vasculitis, and lymphomagenesis.

## Introduction

Thymectomy, a recognized treatment for myasthenia gravis, may have long-term immunological effects by disrupting central tolerance and regulatory T-cell balance.^1^^,^^2^ Such immune imbalance can predispose to systemic autoimmune diseases, including systemic lupus erythematosus (SLE) and Sjögren’s syndrome, which are associated with persistent B-cell activation and cryoglobulin production.^3^^,^^4^ Chronic immune stimulation may promote B-cell clonal evolution from a polyclonal to monoclonal state, increasing the risk of lymphoproliferative disorders such as marginal zone lymphoma.^5^^,^^6^ We describe a distinctive case illustrating this sequence of immune dysregulation leading from thymectomy to overlapping autoimmunity, cryoglobulinemia, and lymphomagenesis.

## Case Report

**Figure 1:**
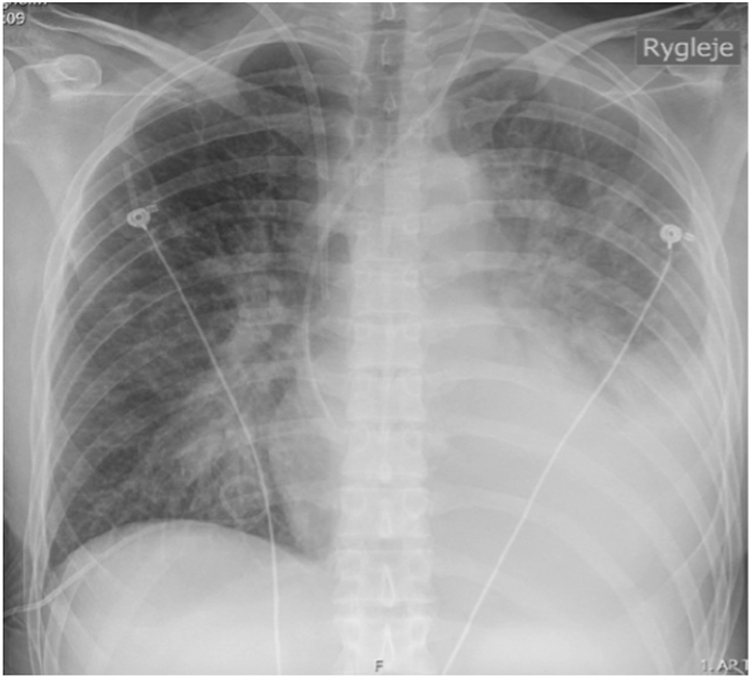
Chest radiograph with Kerley B lines and increased cardiothoracic ratio.

A 39-year-old woman with a 3-week history of diarrhea, vomiting, weight gain of 5 kg, and bilateral lower limb edema was admitted to the hospital. She also reported a 2-week history of fever, malaise, and dry cough. On admission, her vital signs included oxygen saturation 97%, temperature 36.6 °C, blood pressure 177/122 mm Hg, and respiratory rate 20 breaths/min. Arterial blood gas analysis showed metabolic acidosis (pH 7.28, bicarbonate 16 mmol/L, arterial partial pressure of carbon dioxide 33 mm Hg). She presented with tachypnoea and dependent edema. Laboratory investigations revealed plasma creatinine 260 μmol/L (normal, 45-90 μmol/L) (was 88 μmol/L 1 year earlier), hypoalbuminemia 27 g/L (normal, 36-45 g/L), anemia with hemoglobin 4.9 mmol/L (normal, 7.3-9.5 mmol/L), C-reactive protein (CRP) 18 mg/L (normal, <10 mg/L), and urine dipstick protein >3 g/L (normal, <0.15 g/L). A chest radiograph revealed signs of fluid overload and cardiomegaly (Fig 1). A bedside echocardiogram revealed a left ventricular ejection fraction of 10%-15%.

The patient had a complex medical history, including a diagnosis of myasthenia gravis 10 years before the current admission, total thymectomy due to suspected thymoma (which was later ruled out), and a recent diagnosis of marginal zone lymphoma. She had also been under evaluation for SLE. Over the past 2 years, she had experienced pronounced Raynaud’s phenomenon, alopecia, paresthesia of both the upper and lower limbs, migratory joint pain (mainly in the knees and feet), cutaneous changes in the form of pruritic purpura of the lower limbs, intermittent periorbital edema, and a dry cough.

Following transfer to the nephrology department, intravenous methylprednisolone (500 mg/d) was initiated for 3 days. However, within hours, the patient deteriorated abruptly with acute respiratory distress and was transferred to the intensive care unit. She was managed with high-flow oxygen therapy (15-30 L/min, fraction of inspired oxygen 1.0), maintaining her peripheral capillary oxygen saturation at 95%. A pleural drain was inserted, yielding 1900 mL of straw-yellow fluid. Owing to fluid overload and decreased urine output, continuous renal replacement therapy was initiated with 3 sessions of continuous renal replacement therapy and 1 session of hemodialysis, resulting in a total ultrafiltration volume of 8,000 mL. She gradually improved and avoided intubation.

**Figure 2:**
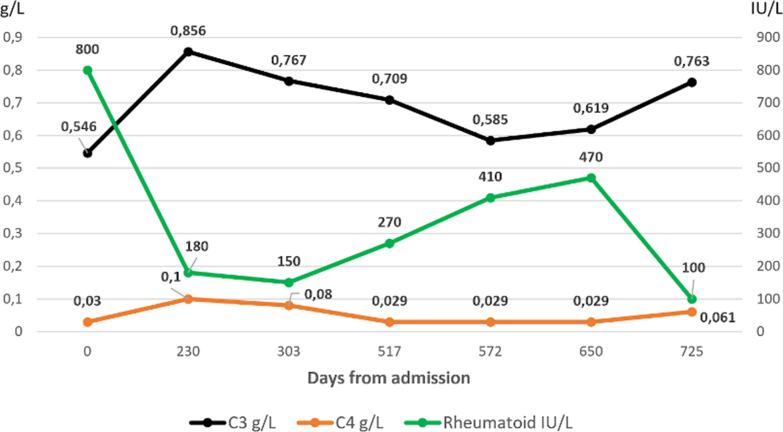
Levels of complement and rheumatoid factor in cryoglobulinemic vasculitis. C3 (black), C4 (green), and rheumatoid factor (orange) from day 0 to day 725. Concentrations of complement system proteins partially recovered, whereas those of rheumatoid factor diminished with immunosuppressive treatment.

Further investigations revealed a urinary albumin-creatinine ratio 763 mg/g (normal, <15 mg/g), elevated IgM 4.50 g/L (normal, 0.39-2.08 g/L), positive IgM monoclonal band, and κ/λ light chain ratio 4.79 (normal, 0.26-1.65). Free κ chains were elevated at 151 mg/L (normal, 3.3-19.4 mg/L), and free λ chains were elevated at 31.5 mg/L (normal, 5.7-26.3 mg/L). Immunoserological tests revealed strongly positive antinuclear antibody, Sjögren’s-syndrome-related antigen A antibodies >240 kU/L (normal, 7.0 kU/L), and Sjögren’s-syndrome-related antigen B antibodies 210 kU/L (normal, <7.0 kU/L). Rheumatoid factor (RF) was markedly elevated at 800 IU/L (normal, <30 IU/L) (Fig 2), anti-double-stranded DNA antibody level was 68 kU/L (normal, <10 kU/L), and her cryoglobulin test was positive; further typing was not available at our laboratory. Complement levels decreased, with C3 at 0.0546 g/L (normal, 0.81-0.57 g/L) and C4 at 0.03 g/L (normal, 0.10-0.40 g/L) (Fig 2). Tests for antiphospholipid antibodies, antineutrophil cytoplasmic antibodies, anti-glomerular basement membrane antibodies, hepatitis B and C viruses, and human immunodeficiency virus were all negative. Electroneuronography confirmed mononeuritis multiplex in the feet.

**Figure 3:**
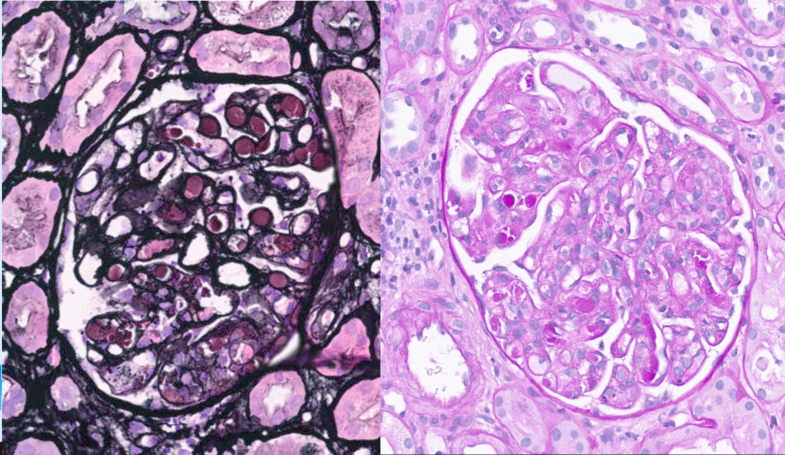
Kidney biopsy (light microscopy and periodic acid–Schiff stain) revealing positive staining of pseudothrombi and endocapillary proliferation, characteristic of cryoglobulinemic vasculitis.

A kidney biopsy revealed numerous periodic acid–Schiff-positive pseudothrombi within the glomerular capillaries, accompanied by endocapillary proliferation. Immunofluorescence revealed deposits of IgA, IgM, IgG, C1q, κ and λ light chains, and C3c within the pseudothrombi. Mild granular C3c staining was observed elsewhere in the glomeruli, without significant immunoglobulin or complement deposition (Fig 3). Electron microscopy was not performed due to insufficient biopsy material.

### Diagnosis and Management of Cryoglobulinemic Vasculitis

Based on the clinical presentation, serology, and, importantly, kidney biopsy revealing extensive pseudothrombi, which are characteristic of cryoglobulin-associated vasculitis, the patient was diagnosed with active cryoglobulinemic vasculitis at the time of admission. High-dose corticosteroid pulse therapy (500 mg intravenous methylprednisolone) was administered for 3 days, followed by 75 mg oral prednisolone daily, which was gradually tapered thereafter by 5 mg every second week. Continuous renal replacement therapy/hemodialysis led to a gradual improvement in cardiac function, which fully recovered after the restoration of renal function, diuresis, and euvolemia. The patient received 2 doses of rituximab (1 g each) on days 11 and 14. After an 18-day inpatient stay, the patient was discharged home. Rituximab therapy was well tolerated, and plasma exchange was not needed.

**Figure 4:**
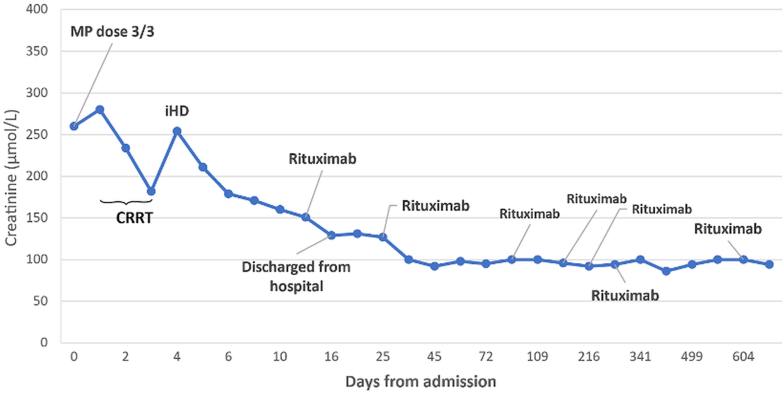
Plasma creatinine measurements. Initial creatinine levels peaked at >260 μmol/L (normal range, 45-90), prompting the initiation of continuous renal replacement therapy (CRRT), followed by intermittent hemodialysis (iHD) during the acute phase. High-dose intravenous methylprednisolone (MP, 3 pulses) was administered early in the course of treatment. The patient was discharged after stabilization of renal function, and plasma creatinine progressively improved after multiple doses of rituximab, which were administered at key time points as indicated. Creatinine values remained stable in the long term, which is consistent with a sustained therapeutic response.

Subsequent follow-up revealed a marked improvement in renal function, with serum creatinine decreasing to 95 μmol/L (normal, 45-90 μmol/L) (Fig 4) and no albuminuria. Detection of cryoglobulin was negative, although complement tests still revealed low C3 0.47 g/L (normal, 0.81-1.57 g/L) and C4 <0.06 g/L (normal, 0.10-0.40 g/L). The steroid dose was further tapered, and 4 additional rituximab treatments were planned upon hematology advice. A positron emission tomography‒computed tomography scan revealed reactive lymph nodes in the liver hilus, with no signs of relapsed lymphoma in the axilla, neck, or groin. Two months after completing rituximab, the patient developed arthralgia, influenza-like symptoms, cough, and joint pain, with signs of vasculitis relapse. Treatment was adapted with titration of the steroid dose to 20 mg and initiation of mycophenolate mofetil (500 mg twice daily). However, mycophenolate was discontinued after 119 days due to gastrointestinal intolerance. Owing to worsening disease activity, including increasing RF levels, complement consumption (Fig 2), and positive cryoglobulins, rituximab was reintroduced with a subsequent attempt at mycophenolic acid (Myfortic) as maintenance therapy. However, owing to persistent symptoms, including paresthesia, Raynaud’s phenomenon, malaise, elevated RF 470 IU/L (normal, <30 IU/L), undetectable CD19, and reduced C3 and C4 (Fig 2), along with positive cryoglobulin, she ultimately remained on rituximab maintenance therapy (1 g every 4-6 months) as part of an autoinflammatory maintenance protocol. Recent investigations revealed that RF decreased to 100 IU/L (normal, <30 IU/L), C-reactive protein <1 mg/L (normal, <10 mg/L), C3 0.763 g/L (normal, 0.811-1.570 g/L), and C4 <0.061 g/L (normal, 0.10-0.40 g/L) (Fig 2). Creatinine level remained almost stable at 92-100 μmol/L (normal, 45-90 μmol/L) (Fig. 4).

### Discussion

This patient presented with a complex interplay of autoimmune and neoplastic diseases. Initially, she was diagnosed with myasthenia gravis, an autoimmune disorder characterized by antibodies targeting the acetylcholine receptor.^7^ Following total thymectomy to alleviate myasthenia gravis symptoms and rule out thymoma (which was ultimately excluded), the patient developed progressive symptoms consistent with SLE and was subsequently diagnosed with this condition >10 years after thymectomy. Thymectomy is known to disrupt immunological self-recognition, potentially leading to an imbalance in regulatory T cells and loss of central tolerance. This mechanism has been implicated as a potential trigger for secondary autoimmune diseases, which in her case manifested with elevated antinuclear antibodies, including Sjögren’s syndrome antigens A and B antibody levels, and positive anti-double-stranded DNA antibodies, aligning with her SLE diagnosis.^1^^,^^2^

Autoimmune diseases such as SLE and Sjögren’s syndrome are often associated with cryoglobulins and can predispose individuals to B-cell lymphomas.^3^^,^^4^ In this case, chronic immune activation due to SLE/Sjögren’s syndrome likely contributed to the proliferation of B-cell clones producing immunoglobulins that bind to IgG, reflected in the patient’s positive cryoglobulin, RF, and elevated IgM levels, as well as a detectable M-component, thus fulfilling the serological criteria for mixed cryoglobulinemia-associated vasculitis.^5^ She also demonstrated complement consumption together with a positive IgM RF. In combination with the presence of an IgM monoclonal band, this serological constellation is indicative of a mixed-type cryoglobulinemia (type II). In contrast, type I cryoglobulinemia, which is typically associated with a single monoclonal immunoglobulin, is usually immunologically inactive, characterized by absent complement consumption, negative RF, and a clinical phenotype dominated by hyperviscosity or acral ischemia rather than systemic inflammation.^8^

This ongoing polyclonal B-cell activation, compounded by the patient’s autoimmune and possibly genetic and environmental factors, appears to have eventually led to the clonal evolution of malignant B cells. This progression culminates in the diagnosis of marginal zone B-cell lymphoma, a well-recognized complication of chronic immune stimulation in autoimmune diseases.^6^^,^^9^ In this patient, potential environmental and genetic predispositions were carefully evaluated and ruled out, suggesting that the observed progression was driven primarily by immune dysregulation related to thymectomy and subsequent autoimmunity.

Immune dysregulation in SLE leads to persistent polyclonal B-cell activation due to chronic exposure to autoantigens and immune complex deposition.^10^ Over time, this excessive immune stimulation may drive certain B-cell clones to outcompete others, resulting in oligoclonal expansion—a state in which a limited number of dominant B-cell populations produce immunoglobulins that contribute to cryoglobulin formation.^11^

As the disease progresses, persistent antigenic stimulation and inflammatory cytokine environments create selective pressure favoring monoclonal evolution, where a single dominant B-cell clone begins to proliferate unchecked.^12^ The presence of an M component in the patient’s serum, a hallmark of monoclonal gammopathy, suggests that the immune system has shifted from a reactive immune response to an early neoplastic process.^13^
